# Ours to eat and own: assessing the feasibility of a cooperative meal-kit service to improve food access

**DOI:** 10.1017/S1368980023002884

**Published:** 2023-12-21

**Authors:** Joelle N Robinson-Oghogho, Joel Gittelsohn, Janice Bowie, Lois Dankwa, Roland J Thorpe

**Affiliations:** 1 Johns Hopkins Bloomberg School of Public Health, Department of Health Behavior and Society, Baltimore, MD 21205, USA; 2 Johns Hopkins Bloomberg School of Public Health, Department of International Health, Baltimore, MD, USA; 3 Johns Hopkins Bloomberg School of Public Health, Department of Health Policy and Management, Baltimore, MD, USA; 4 Hopkins Center for Health Disparities Solutions, Johns Hopkins Bloomberg School of Public Health, Baltimore, MD, USA

**Keywords:** Meal kits, Food access, Dietary interventions, Feasibility study, Pilot, Community-based

## Abstract

**Objective:**

Although typically serving higher income and younger demographic groups, meal-kit subscription services have the potential to improve food availability and dietary quality in communities experiencing low food access due to systemic discrimination. This study describes the development and characteristics of a pilot community-led meal-kit service (SouthEats) and evaluates key implementation outcomes of adoption, acceptability, and feasibility among households experiencing less income.

**Design:**

We utilised a mixed methods study design, including data from administrative records, customer surveys and worker interviews. Thematic qualitative analyses and descriptive quantitative analyses were conducted to illuminate the characteristics and extent the pilot meal-kit service was adopted, acceptable, and financially feasible among the target populations.

**Setting:**

The study took place in Washington DC, USA.

**Participants:**

Study participants included SouthEats consumers (*n* 35) and workers (*n* 3).

**Results:**

During the pilot period, sixty-seven community members signed up for the meal-kit service, with 52 % making recurring purchases. Our results suggest that the meal-kit service is acceptable among people living in low food access areas. Our feasibility analysis indicates that, although not without challenges, the SouthEats model could be financially feasible.

**Conclusion:**

These preliminary insights can inform the scalability and potential replication of this service and provide foundational evidence for an approach that may be used to improve food access.

Home food delivery has been one approach to improve access to healthy food for people experiencing challenges due to their neighbourhood environment and/or personal physical limitations. Most long-standing home food delivery programmes such as Meals-on-Wheels, medically tailored meals and commercial weight-loss meal plans cater to individual use or target people with specific existing health conditions. However, individuals often live in households with a multitude of co-habitants. Meal-kit services are a relatively new food procurement business model, typically designed for multi-person households. Although consumers of meal-kit services have been characterised as high-income young adults^(1)^, meal-kit services may also be a viable solution for improving access among people who are economically constrained^(2,3)^. Existing studies on the acceptability of meal kits in various populations have found them to be acceptable in various populations, including among low-income populations^(2,4–6)^.

Two published studies implemented community-based meal-kit services; yet, each of these interventions either relied on donated staff time or materials to provide meal kits at an affordable price^(2,3)^. These unaccounted costs make it difficult to assess if community-based meal-kit services would be feasible in a real-world context. Additionally, existing studies do not provide details describing the key elements or barriers that would or would not make such approaches possible. Running a food business is difficult. Food businesses are known to have small profit margins^(7)^, approximately 60 % of independent restaurants fail within 3 years of opening, and it typically takes 2 to 3 years for a business to become profitable^(8,9)^. The current food system is largely exploitive of workers, consumers and the environment. However, the field of public health intersects with each of these sectors and is well positioned to make a case for businesses and governments to leverage resources that facilitate alternative food models that are not solely based on a profit-driven approach. Insights on the development and sustainability of meal-kit services created by and for disinvested populations could help practitioners, researchers, and policymakers better understand how food businesses may help advance health and social equity through their practices and inform the scalability and replicability of future models.

Guided by the conceptual framework and taxonomy for implementation research developed by Proctor et al.^(10)^, this study focuses on understanding the intermediate implementation outcomes most salient to the early phase of the SouthEats meal-kit service, typical of pilot and feasibility studies^(11,12)^. Using administrative records, customer surveys and worker interviews, we addressed the following research aims:

1. Identify key elements of the SouthEats meal-kit service.
2. Examine the extent to which the SouthEats service was adopted and found to be acceptable among households with low to middle incomes (i.e. ≤ 80 % area median income) in Washington DC.
3. Evaluate the financial feasibility of the SouthEats meal-kit service model.

## Methods and materials

### Study setting and context

This pilot intervention and study occurred in Washington DC. Like many US cities, the geography of Washington DC is economically and racially divergent. Based on data from the 2020 census, Washington DC has a high level of racial segregation^(13)^. Of DC’s eight Wards, Ward 7 and Ward 8 are home to the highest proportion of Black residents in the city, yet also have the lowest average income, and the highest burden of chronic conditions^(14,15)^, Further, the economic inequities by race and place are stark. In 2021, the overall median household income for all district residents was $93 547. However, for White residents it was $150 563, compared with $51 562 for Black residents^(16)^.

Corresponding to these economic disparities, the food environment in DC’s Ward 7 and Ward 8 is also inequitable. In 2016, there were only three full-service grocery stores in Wards 7 and 8 for over 148 000 residents, compared with nine full-service grocery stores in Ward 3 alone^(17)^. The lack of healthy food retail and grocery options in this region of the city disproportionately and adversely impacts people of colour. Various community mobilisation efforts have voiced the need for healthy food options in these Wards and galvanised to develop innovative community-driven solutions to address this issue^(18,19)^. The SouthEats meal-kit service is one example. The SouthEats pilot occurred within this context as well as during the COVID-19 pandemic, which potentially compounded the situation of those already experiencing inequitable access to food.

### Intervention

SouthEats is a worker-owned cooperative food business developed by DC residents living in Wards 7 and 8, seeking to provide affordable, locally sourced, culturally relevant, healthy meals to communities that have historically been excluded from accessing healthy food options^(20)^. The initial conception and seed funding for the project were provided by the Robert Wood John Foundation (RWJF), after three of the founding members completed the RWJF Culture of Health Leaders Program^(20)^. The target areas for the implementation of the SouthEats pilot were neighbourhoods in Wards 7 and 8, with a specific focus on low- and middle-income households (i.e. ≤ $74 837) and recipients of the National Supplemental Nutrition Assistance Program (SNAP).

Meal-kit services vary in the degree of preparation required. Some services provide raw groceries that require chopping or additional ingredients, while others are partially prepped or fully prepped. Meals from SouthEats are fully prepped to include pre-seasoned, cut/chopped and pre-portioned uncooked food items that do not require any additional ingredients for the meals to be prepared. Like other meal-kit services, customers select three meals to receive once a week, from a list of five menu options that change each week. Menu items were developed by SouthEats team members and evaluated by a nutritionist consultant. The price of the weekly meal kits started at $25 for the half-size option (i.e. three meals with two servings) and $45 for the full-size option (i.e. three meals with four servings). However, during the pilot period, the SouthEats team increased the prices of the meal kits to $38 and $75 for the half-size and full-size options, respectively. Along with their meals, customers were provided with a brief 5- to 7-step cooking instructions sheet for each meal. The estimated cooking time for meals ranged from 25 to 45 min.

### Data sources

Data collected for this study come from three sources: survey data from SouthEats customers; semi-structured interviews with SouthEats workers; and historical and administrative data from SouthEats. Survey data were collected online from SouthEats customers who self-elected to join the study. All customers were invited to join the study at the time of purchase. Eligible participants were adults, 18 years or older, who indicated they were responsible for at least 50 % of the cooking or food shopping in their household and were not receiving any other commercial meal-kit service. Participants were recruited between July 2021 and December 2021 and completed baseline, midpoint, and endpoint surveys over 8 weeks. Each survey took approximately 25 min to complete. Participants were provided with a $25 incentive after each survey completion. An additional incentive of $20 was provided for completing all three surveys.

Qualitative interviews were conducted with three out of the four current SouthEats workers and led by a trained research assistant in July 2022. Of the three workers interviewed from SouthEats, one had been with the cooperative since its inception in 2016, one since the initial launch of the meal-kit service in 2019, and the other interviewee had been a worker with the cooperative for 4 months at the time of the interview.

We also gathered historical documents produced by SouthEats during the initial development phase (i.e. 2016–2019) and administrative data on website analytics (i.e. weekly sales and website visits) collected from July 2021 to December 2021 via the web hosting service wix.com.

### Measures

#### Aim 1: assessing SouthEats key elements

To address the first aim of identifying the intervention’s key elements, we developed an intervention logic model using the aforementioned qualitative data. Logic models have been consistently noted as useful tools to illuminate intervention components and inform subsequent evaluation plans^(21,22)^. To identify key elements of the SouthEats model, six *a priori* codes reflecting the basic components of a logic model and programmatic theories of change were used. The codes were defined as follows: (1) *Challenges:* what makes it difficult to implement or operate the SouthEats model as desired; reasons why certain goals may have not been achieved; (2) *Goals, Values, Vision:* explicit and implicit goals, intentions, vision, and values of SouthEats. Underlying principles, standards or aspirations that guide the development or implementation of SouthEats; (3) *Key Inputs*: tangible and intangible elements that go into the development and operation of SouthEats; things needed for the success and continuation of the SouthEats model; (4) *Mechanisms*: theory of change; programme theory; reasoning about components of the SouthEats model or way of operating that allows for the accomplishment of desired goals; (5) *Lessons Learned*: knowledge, understanding or information gained thus far; new information learned from the development and implementation process; and (6) *Implementation*: the extent SouthEats is, or is not, delivered as intended; instances when programme components are, or are not, provided or retrieved by customers satisfactorily.

#### Aim 2: assessing adoption and acceptability

Our measures of adoption and acceptability were selected based on examples in the implementation science literature^(10)^ and consultation with SouthEats team members about which information was available and feasible to collect.

To assess aim 2, we used customer survey data and SouthEats administrative data sources. Adoption (i.e. the initial decision to try or use a new intervention)^(23)^ was assessed via measures of interest, retention and reasons for use. Interest and retention were captured using three measures: (1) number of unique visitors to the SouthEats website during the pilot study period (i.e. July 2021–December 2021), (2) number of customers during the pilot period and (3) proportion of customers who purchased a 1-week trial plan compared with the proportion of customers who purchased multiple weeks of SouthEats meals. We examined survey data to explore reported reasons for use among SouthEats customers. Survey participants were asked the open-ended question ‘Why did you sign-up for SouthEats Meals?’

To assess acceptability (i.e. perceptions among consumers that the intervention is agreeable, palatable or satisfactory)^(10,23)^ we used measures of liking and continued use intentions among participants in the SouthEats customer study in the midpoint and endpoint surveys. Liking was assessed using two questions that asked study participants to select the meals they received from SouthEats and rate on a scale of 1–5 (i.e. disliked a great deal – liked a great deal) the taste and visual appeal of the selected food items. Since SouthEats provided over sixty different menu items via a changing weekly menu, during the pilot study period, a selection of twenty-five items was included in the midpoint and endpoint surveys to evaluate liking. For each meal category (i.e. beef, poultry, seafood and vegetarian), we calculated the combined mean taste and appearance scores using the average score for each meal. Participants were also asked at midpoint and endpoint data collection periods, about their intention to continue using the meal-kit service via the question, ‘Based on your experience with the SouthEats meals so far, how likely would you be to continue ordering meals from them after this trial period ends?’ We created a binary variable, where ‘very likely’ and ‘likely’ were coded as Yes, while ‘Not Sure, Not Likely, and Very Unlikely’ were coded as No/Not Sure.

Additionally, we collected information on the demographic and behavioural characteristics of participants in the SouthEats customer survey. Characteristics include participants’ age, race and ethnicity (i.e. African American; Afro Caribbean or African; Hispanic; White; East Asian; South Asian or Pacific Islander, Native American/First Nation; Other), educational attainment, household income, household size, presence of children aged 17 years and younger in the household, zip code of residence, SNAP participation, previous use of meal-kit services, and food values. Food values were assessed by asking participants to rank from a list of eleven choices the most important aspects when purchasing food. Food value options were price, quality, freshness, time/how long it will take, if other members of my household like it, familiarity/something I have eaten before, taste, health effects, supporting a small business or under-represented business, effects of product on the environment and effects of product on workers. These food values were included to reflect constructs of existing scales and frameworks on food choice motives^(24–27)^ and adapted for relevance to our study population. We report on the values ranked as the most important and the proportion of respondents that ranked them as such.

#### Aim 3: assessing financial feasibility

Feasibility refers to the extent an intervention can be carried out in a particular setting^(23)^. In this case, we were particularly interested in the financial feasibility of the SouthEats model among the target population. To assess the financial feasibility of SouthEats, we used administrative records to estimate the cost of producing one meal-kit unit, consisting of one beef, one seafood and one poultry meal. The cost calculations accounted for ingredients, labour, commercial kitchen rental fees and other overhead costs. To account for economies of scale, production cost was calculated for producing varying quantities of meal kits per week (i.e. units of full-size orders). We compared this information to responses in the customer survey that asked, **‘**What is the most you would pay to receive 3 prepped meals (i.e. packaged, seasoned, portioned, uncooked) that serve 4 people (12 servings total) each week?’ We explored if the estimated cost for providing the meal-kit service aligns with what participants reported being willing to pay.

### Analysis

We utilised qualitative data analyses to elucidate the key elements of the SouthEats intervention. Worker interview transcripts and historical documents (i.e. web pages and grant applications) were coded using the codebook described in the measures section above. Qualitative analysis software, Atlas.ti version A8, was used to organise qualitative data and codes. The text corresponding to each code was then examined using the one sheet of paper method as an axial coding approach, as described by Zeibland et al.^(28)^ These data were used to construct a variation of an intervention logic model that reflected SouthEats team member’s articulation of what they hoped to accomplish with SouthEats, the key inputs and activities that were needed, and the rationale for conducting the activities. The logic model was reviewed and validated by the SouthEats worker-owners. This analytic approach aligns with what has been described in methodological texts for case study analyses and programme evaluation^(21,29)^.

For analyses related to aims 2 and 3, we conducted descriptive quantitative analyses of customer survey and website administrative data for key study outcomes of adoption (i.e. interest and retention), acceptability (i.e. liking and continued use intentions) and financial feasibility (i.e. willingness to pay *v*. cost to produce). For the construct of adoption, customer responses to the short-answer reasons for use survey question were open-coded, categorised into common themes and then quantified. We also compared the characteristics of SouthEats survey participants who completed all study surveys to those who did not, using *t* test for continuous variables and Fisher’s exact chi-squared test for categorical variables, at a 95 % CI.

## Results

### Aim 1: key elements of SouthEats

**Fig. 1:**
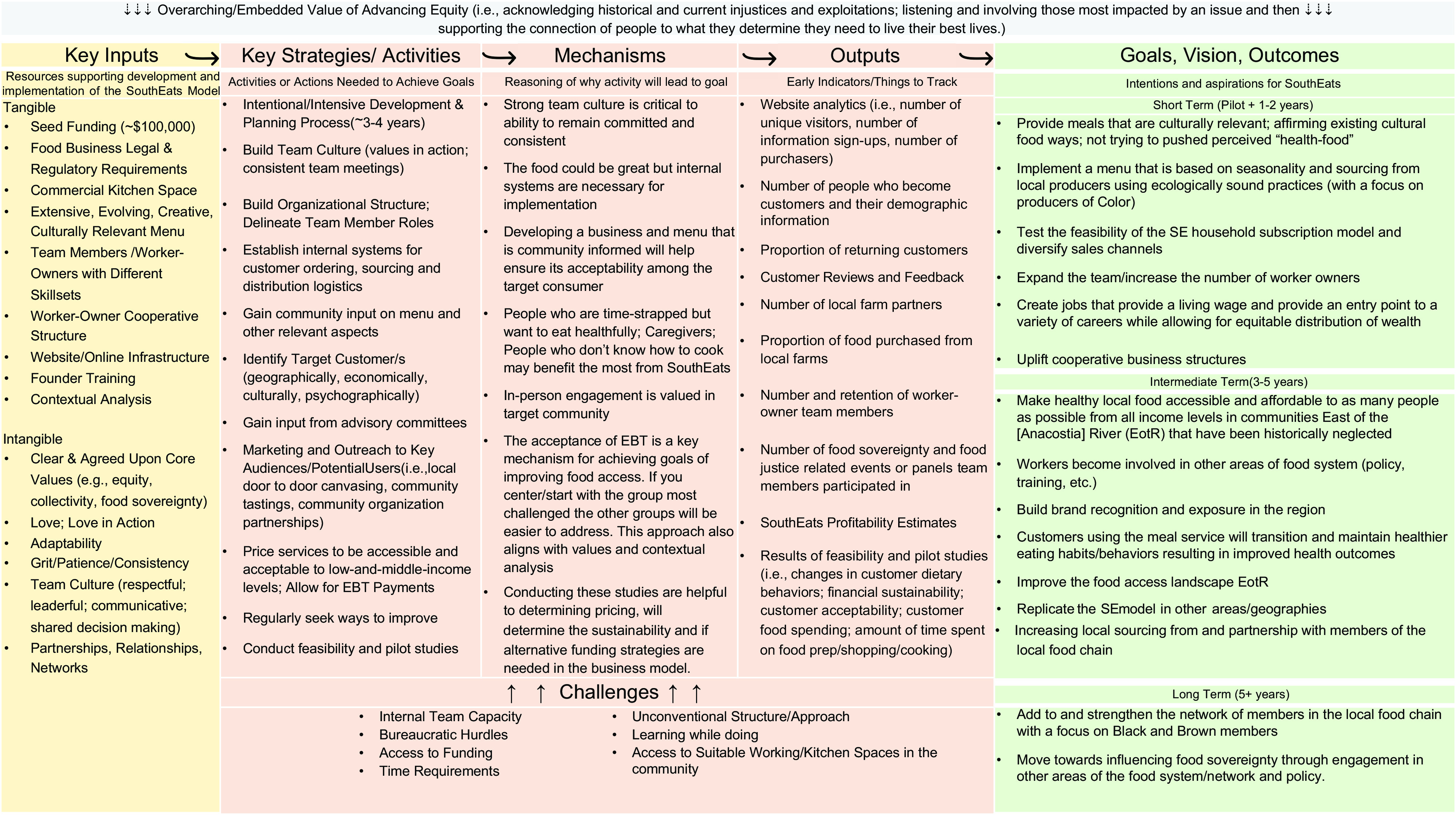
SouthEats meal-kit service logic model

The logic model for the SouthEats meal-kit service, highlighting the goals, key inputs, strategies, and mechanisms involved in its development and implementation, is displayed in Fig. 1. The SouthEats meal-kit delivery service attempts to address factors, such as inequitable neighbourhood access to healthy foods, affordability, time constraints that limit home cooking and individual cooking skills, by delivering locally sourced ready-to-cook meals that are accessible to households participating in SNAP. This is reflected in some of the goals outlined in the logic model, such as improving the food landscape of the community and supporting customers’ transition to and maintaining healthier eating habits through culturally relevant meals. Accomplishing the organisational goals required various tangible and intangible key inputs. Tangible inputs include seed capital made available through grant funding, commercial kitchen space and website infrastructure. However, workers also discussed important intangible inputs such as love and adaptability, illustrated in the following quote.

*‘….But I honestly think love, that is the engine. If there wasn’t love, it could function, for sure it could totally function, it would just be very different. Right now, SouthEats, the meals that we provide on a subscription basis are priced so that they’re as accessible to as many people as possible. And there’s no reason to do that unless you care, unless you love the people. And I think SouthEats, I mean we love. It’s our people, you know.’* – Quote from SouthEats Worker Interview

An additional, key component was the worker-owned cooperative organisational structure. A worker-owner cooperative is a business that is owned and controlled by its workers. For SouthEats, this business structure represents a deliberate attempt to disrupt the differences in power and social positions that allow disparities in wealth and health to accumulate, by incorporating equal decision-making power and ownership among workers. Here, all workers, regardless of role, were paid the same living wage of $17/h. As a key activity, this presented both benefits and challenges. For example, the business’s internal systems and team culture of collectivity and equity were heavily influenced by its worker-owner cooperative structure, while the limited capacity of the small worker-owner implementation team was a challenge that impacted the ability to expand and gain new customers, and contributed to some inconsistencies in when weekly menus were posted to the website, and the labelling of food items for customers.

Additional key activities were an intensive development process, which included a 3-year design and conceptualisation phase and a formative development phase consisting of community input, the formation of an advisory committee, and a smaller testing period with 10 families from the target community. Community involvement in the development of the menu items and business, establishing internal structures, and leveraging existing community partnerships and relationships, all informed the business format for the pilot phase.

Our overall qualitative analysis also revealed equity as the predominant value of the SouthEats organisational model, as seen in the top box on Fig. 1. The aspiration to implement a model that advances equity both internally and externally was reflected in interviews and documents. For example, the decision to structure SouthEats as a worker-owner cooperative, to prioritise affordability and multiple payment methods, and to focus on serving communities in under-resourced areas, and the goal of partnering with and sourcing from local farmers of colour are rooted in a definition of equity that acknowledges historic and ongoing injustices and exploitations.

General challenges mentioned by SouthEats workers included the bureaucratic hurdles related to obtaining required local regulatory licences and certifications for this type of food business, and the lengthy and time-intensive development process, which were both potentially exacerbated by the COVID-19 pandemic.

### Aim 2: SouthEats adoption and acceptability

As mentioned, the primary source of information used to assess the implementation outcomes of adoption and acceptability were gathered from customer surveys. Participant characteristics from the SouthEats online customer survey are shown in Table 1. Our study sample consisted of thirty-five participants who completed the baseline survey, of those, twenty-three participants completed all three baseline, midpoint and endpoint surveys. The average age of completer survey participants was 42 years (s d = 12·3). Nearly 90 % of participants identified as Black. Among baseline participants, 63 % of baseline lived in the target areas of Ward 7 and 8, 17 % lived in the neighbouring area of Prince George’s County MD and 17 % lived in other areas of Washington DC. Over 30 % of participants were SNAP recipients. Forty-five per cent of survey participants had household incomes less than $75 000. Sixty-nine per cent of baseline survey participants never used a meal-kit service previously. Health, quality and freshness were the top-ranked factors of importance for purchasing foods, among SouthEats customers participating in the study. The characteristics of customers who completed all pilot study surveys did not differ significantly from those who did not complete all of the surveys (Table 1).

**Table 1:** Descriptive characteristics of 2021 SouthEats pilot study survey participants

	Customer survey completer *v*. non-completers									
	Total participants *n* 35			Non-completers *n* 12			Completers *n* 23			
	%	Mean	sd	%	Mean	sd	%	Mean	sd	*P*-value
Age, mean, (sd)		42·4	11·4		41·7	9·9		42·9	12·3	0·77
Race, %										1·00
Black only and Black + another	88·6			91·7			87·0			
White or native	8·6			8·3			8·7			
Missing/no response	2·9			0·0			4·4			
Educational attainment, %										0·97
High school grad or GED	5·7			0·0			8·7			
Some college or tech school	22·9			25·0			21·7			
College grad	31·4			33·3			30·4			
Grad school or terminal degree	37·1			41·7			34·8			
Missing/no response	2·9			0·0			4·4			
Household income, %										0·28
Less than $75000	45·7			33·3			52·2			
Greater or equal to $75000	48·6			66·7			39·1			
Missing/no response	5·1			0·0			8·7			
Household size, %										0·76
1–2 members	40·0			50·0			34·8			
3–5 members	48·6			41·7			52·2			
6 or more members	8·6			8·3			8·7			
Missing/no response	2·9			0·0			4·4			
Children in household, %										0·74
Yes	54·3			50·0			56·5			
SNAP participation, %										0·68
Yes	34·3			25·0			39·1			
No	54·3			58·3			52·2			
Missing/no response	11·4			16·7			8·7			
Geography, %										0·09
DC Ward 7 and 8	62·9			41·7			73·9			
DC other	17·1			33·3			8·7			
PG county, MD	17·1			25·0			13·0			
Missing/no response	2·9			0·0			4·4			
Ever used meal kits, %										0·71
Yes	31·4			25·0			34·8			
No	68·6			75·0			165·2			
Missing/no response	0·0									
Food values – most important, %										
Health	22·9			16·7			26·1			
Quality	20·0			8·3			26·1			
Freshness	17·1			16·7			17·4			
Price	14·3			25·0			8·7			
Taste	11·4			16·7			8·7			
Time	5·7			8·3			4·4			
Household members	5·7			8·3			4·4			
Familiarity	2·9			0·0			4·4			

*n,* number of participants; SNAP, Supplemental Nutrition Assistance Program.

*P*-values indicate if a statistically significant difference exists between participants who completed the baseline survey but did not complete subsequent surveys (*n* 12) and those who completed all three surveys (*n* 23), using paired *t* test for continuous variables and Fisher’s exact chi-squared test for categorical variables, at a 95 % CI.

#### Adoption

Between July 2021 and December 2021, there were 1387 new unique visitors to the SouthEats website. A total of 127 website visitors created an account to allow them to receive updates, send messages to the SouthEats team or place orders. During the pilot study period, sixty-seven new customers purchased meals from SouthEats. This indicates that approximately 9 % of people who visited the website created an account and 5 % purchased meals. To assess retention, we examined the proportion of recurring customers. Of the sixty-seven SouthEats customers, 48 % (*n* 32) were one-time purchasers receiving meals for 1 week and 52 % (*n* 35) purchased a subscription or multiple times, to receive meals for more than 1 week. Of the thirty-five survey participants, thirty-two responded to the open-ended question asking why they chose to receive meals from SouthEats. We identified six common reasons for use: trying something new, health, supporting minority business, affordability, recommended or gifted, and saving time. The most frequently mentioned reasons for use were to support a minority business, which was mentioned in nineteen responses, followed by saving time, mentioned in ten responses, and curiosity/trying something new, mentioned in nine responses.

#### Acceptability

Liking and continued use intentions measures were used to assess acceptability. On average, liking in terms of taste and visual appeal were positive for the beef and poultry meals, but more variable for the fish and vegetarian meals. On a scale of 1–5, the combined average rating for the two beef meals was 4·36 for taste and 4·14 for appearance. For the nine poultry meals, the combined average customer ranking for taste was 4·17 and 4·39 for appearance. Of the eight seafood meals, the combined average customer score for taste was 4·0 and 3·91 for appearance. Finally, the combined average score for the six vegetarian meals was 4·31 for taste and 3·95 for appearance. Regarding continued use intentions, in the midpoint survey, 65·2 % of the twenty-three participants who completed all data collection surveys indicated that they were likely to continue purchasing meals from SouthEats, and this increased to 73·9 % at the time of the endpoint survey. At both time points, 21·7 % of participants indicated they were unsure or unlikely to continue to purchase meals, with the remaining participants not responding.

### Aim 3: feasibility

**Fig. 2:**
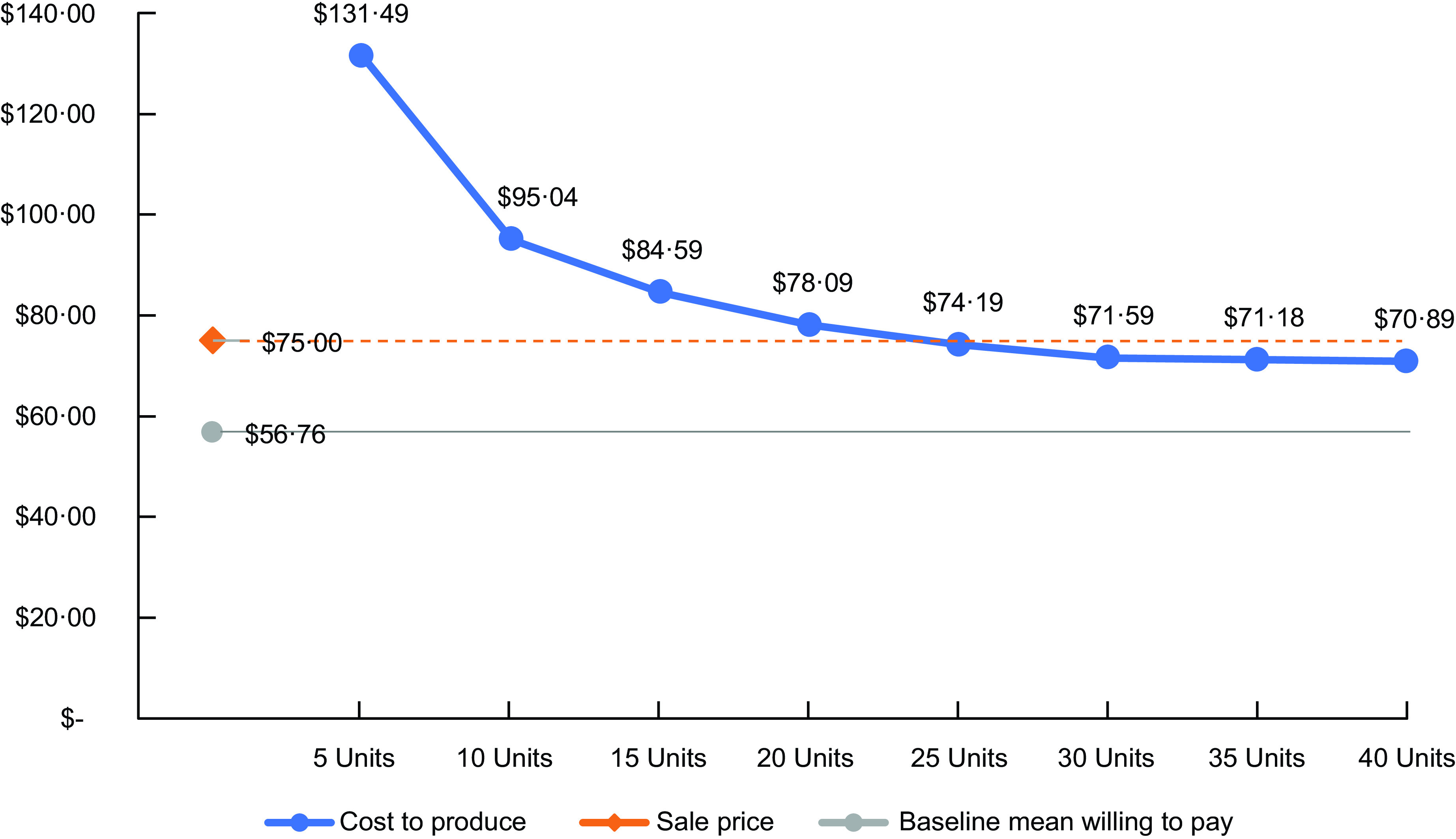
SouthEats financial feasibility estimates for 2021 pilot study. Units refer to the number of full-size SouthEats meal-kit orders produced in 1 week (1 unit contains three meals with four servings per meal). Cost to produce reflects the estimated US dollar amount required to produce 1 unit of SouthEats meals. Sale price reflects how much 1 unit of SouthEats meals were being sold at the time of this pilot study. Baseline mean amount willing to pay reflects the average amount SouthEats customers who participated in the pilot study survey indicated they would be willing to pay for 1 unit of SouthEats meals.

Our analysis of the financial feasibility of SouthEats among the target population suggests some discrepancy between the cost to produce the meal kits and what study participants indicated they would be willing to pay. At the time of the baseline survey, the average price participants indicated they would be willing to pay for the service was $57·00. The estimated cost to produce one meal kit ranged from $131·50 to $70·85 depending on the volume of units being produced. The sale price for the meal kits was $75·00. Our results indicate that a minimum of twenty-five subscribing customers are needed to sustain the production of the meal kits at the current sale price (Fig. 2).

## Discussion

This pilot study sought to describe key elements of the SouthEats meal-kit service and explore its adoption, acceptability, and financial feasibility in Washington DC. Our analyses of SouthEats documents administrative data, customer surveys and worker interviews provided insights on the key developmental inputs, including funding, space, website infrastructure, team members, partnerships and shared contextual analysis. Core values such as equity, collectivity and food sovereignty along with a non-hierarchical business structure were noted as additional key inputs. During the pilot period, the meal-kit service was generally acceptable, with just over half of SouthEats customers purchasing recurring orders and survey participants providing positive ratings of the taste and visual appeal of the meals. However, our analysis raises some additional questions about the financial feasibility and subsequent sustainability of the current SouthEats model.

Reasons for using the meal-kit service among study participants aligned with findings from other studies regarding the desire to try something new, health promotion and time savings^(30,31)^. However, SouthEats users’ additional motivations of supporting a minority business and affordability suggest these may also be important features in this target population. Among leading meal-kit companies, the average price per serving ranges from $9·99 at Blue Apron to $4·99 at Every Plate, with most companies at the higher price point^(32)^. The SouthEats price per serving is $6·25, which places it on the more affordable end of the spectrum. As price and affordability are consistently indicated as a barrier to trying meal kits, or the reason for discontinued use^(33)^, ensuring affordability is critical to the viability of this specific service and its potential to address food access barriers among lower-income populations.

Our analysis of the financial feasibility of the SouthEats model indicated that the amount customers would be willing to pay for the service was lower than the cost to provide the service. However, this finding has several caveats. First, during the pilot period, SouthEats increased the price of the meal kits to $75·00, an amount exceeding the willingness-to-pay survey response options. Since new customers continued to purchase meal kits after the price increased, it is plausible that the $75·00 price was acceptable among the target population. Second, in our study, the willingness-to-pay question was only asked in the baseline survey, before participants received their meal kits. The only other study we are aware of that examined willingness to pay for a similar meal-kit service among low-income African Americans found that participants indicated being willing to pay a lower average price of $74·03 at baseline and later indicated being willing to pay $88·61 after receiving the meals^(2)^. Together, this suggests the $75·00 price point would be acceptable among the target population. To move towards financial sustainability, we estimated SouthEats would need a minimum of twenty-five weekly customers purchasing full-size meal kits. However, increasing the meal-kit price may provide additional support for marketing and administrative tasks that the workers-owners expressed having limited capacity towards.

This study examined a food business model with outcomes relevant to public health. However, it is important to note that SouthEats is a for-profit business and not a charity-based model. The worker-owned cooperative structure was a key element for SouthEats that aligned with the business’s equity-driven values. It is possible that the cooperative business structure impacted our financial assessment. However, this is less likely as our analysis accounts for labour costs.

This study has some limitations. First, the customer survey did not capture the full SouthEats customer base reached during the pilot phase. Although we compared participants who completed the survey to those who did not, it is possible that the individuals who participated in the survey were different or had different experiences with the meal-kit service than customers who did not participate. Additionally, qualitative data from customers could have enhanced our understanding of perceptions of the SouthEats meal-kit service. Finally, as mentioned above, our assessment of the financial feasibility of this meal-kit model only collected willingness-to-pay information at baseline with four pre-established price categories (i.e. $45, $50, $60 and $70 each week). Using a more sensitive measure of willingness to pay may have more accurately captured this construct. Additionally, after completing the financial analyses for this study US food prices increased considerably^(34)^; likely increasing the meal-kit production cost. Those seeking to continue or replicate a similar meal-kit service could consider scaled or income-based pricing models to ensure affordability for lower-income earners.

Nonetheless, our analysis provides useful information about the potential utility of a community-based approach for providing a meal-kit service to improve food access. While this study was not designed to assess the extent SouthEats was able to realise its intermediate and long-term goals, our qualitative analyses identified key elements, resources and development processes needed for implementation, which may help inform the replicability of the SouthEats approach. We found that the predominant value that underpins the SouthEats organisational model is equity. This overarching value influenced all aspects, decisions, approaches, and goals articulated and implemented by SouthEats. Those aiming to replicate or learn from this pilot should consider how the intangible components of SouthEats can be transferred and the implications of including or exclusion of certain elements.

Another strength of this study was that it evaluated acceptability in a real-world setting where participants paid for the meal-kit service. This helped to reduce potential food waste that could be caused by participants not picking up the meals if they were provided at no cost, allowed participating households the opportunity to stop using the service if they choose and provided better insights into the potential sustainability of this type of community-based meal-kit service, by attempting to account for the true implementation cost. As cost is a key factor impacting both consumer food choices and intervention viability, providing free or drastically discounted services with the uncertain availability of sufficient resources to maintain the subsidised cost is unlikely to retain users or result in sustained interventions. As profit-driven food corporations are often associated with poor health^(35,36)^, this study highlights a business model attempting to advance public health aims while operating within the capitalist reality.

Meal-kit service may provide an intermediate solution to increase the frequency of home cooking and improve dietary behaviours. Additionally, as people increased at-home cooking during the pandemic^(37)^ due to restrictions on in-person dining and gathering, the use of meal-kit services also increased among both people living and not living in low food access areas^(38,39)^. The COVID-19 pandemic also accelerated the implementation of the SNAP online purchasing programme to allow the millions of US households participating in SNAP^(40)^ to use their SNAP benefits to purchase grocery items online^(41,42)^. During our 6-month pilot period, 35 % of SouthEats customers purchased the meal kits using their SNAP benefits. This provides further evidence to suggest that meal kits, specifically community-based models, could be considered in the catalogue of approaches to promoting healthful dietary behaviours among this population. Further research examining the extent to which utilisation (i.e. consumption, frequency and duration) of meal-kit services influences dietary behaviours and addresses barriers to healthful food consumption is warranted.

### Conclusion

Grassroots community-led interventions may present an approach to addressing issues of inequity rampant in the food system. This study of the SouthEats meal-kit service provides an example of such an approach. The information gleaned from this feasibility study could be used to (1) inform the scalability of this service, (2) help inform which food services become eligible for purchase using SNAP/EBT benefits and/or (3) serve as a model for how grassroots businesses can achieve multiple social and health improvement goals.
